# Assessing mobile phone attachment: a psychometric study in the Portuguese young adult population

**DOI:** 10.1186/s41155-026-00400-6

**Published:** 2026-06-10

**Authors:** Ângela Leite, João Marôco

**Affiliations:** 1 Centro de Estudos Filosóficos e Humanísticos, Catholic University of Portugal, Braga, Portugal; 2 Universidade Lusófona, Lisbon, Portugal

**Keywords:** Attachment, Problematic behavior related to mobile phone, Burden, Refuge, Young adult attachment to phone scale

## Abstract

**Background:**

The Young Adult Attachment to Phone Scale (YAPS) is a 6-item self-report measure assessing emotional attachment to smartphones across two dimensions: using the phone as a source of comfort (Refuge) and experiencing it as burdensome (Burden). This study aimed to evaluate the psychometric properties of the YAPS in a Portuguese young adult sample.

**Methods:**

Participants were 648 Portuguese young adults (aged 18–35). We examined factorial structure via confirmatory factor analysis, reliability, convergent and discriminant validity, and measurement invariance across gender and education level. Given the ordinal nature of the items, analyses employed the DWLS estimator.

**Results:**

The two-factor structure was confirmed with excellent model fit (CFI = 0.990, RMSEA = 0.069). Internal consistency was good for Refuge (ω = 0.83) and acceptable for Burden (ω = 0.74). Both factors demonstrated convergent and discriminant validity. Full measurement invariance was established across gender, and partial invariance across education levels. Latent mean differences emerged by education but not by gender.

**Conclusion:**

The Portuguese YAPS is a valid and reliable instrument for assessing phone-specific attachment in young adults. Findings support its use in research and practice, while highlighting the need for further refinement of the Burden dimension. The scale offers a culturally appropriate tool for understanding technology engagement in Portugal's highly connected youth population.

**Supplementary Information:**

The online version contains supplementary material available at 10.1186/s41155-026-00400-6.

## Introduction

The proliferation of smartphones has fundamentally altered how individuals interact, communicate, and experience daily life. While these devices offer significant functional and social benefits, growing concern surrounds the psychological and behavioral consequences of excessive or maladaptive use, particularly among young adults (Harris et al., 2020; Kuss et al., 2018). In seeking to understand the mechanisms underlying problematic smartphone use, researchers have increasingly turned to attachment theory as a relevant framework (Konok et al., 2016; Trub & Barbot, 2016).

Originally developed to explain close interpersonal relationships, attachment theory posits that individuals develop characteristic patterns of relating to others - secure, anxious, or avoidant - based on early interactions with caregivers (Bowlby, 1969). These attachment styles persist into adulthood and influence not only romantic and platonic relationships but also interactions with objects and technologies (Konok et al., 2016; Trub & Barbot, 2016). Recent studies demonstrate that individuals with high interpersonal attachment anxiety are especially prone to problematic mobile phone use, often seeking reassurance and maintaining constant connection to compensate for unmet attachment needs (Jiang & Zhao, 2016; Liang et al., 2021; Wang & Xuang, 2024). Conversely, those with high interpersonal attachment avoidance may develop maladaptive patterns through different pathways—using the phone as a barrier to create emotional distance, control social availability, and avoid intimate face-to-face interactions, which can reinforce feelings of loneliness (Gritti et al., 2023; Sun & Miller, 2023; Wang & Xuang, 2024). These findings provide the theoretical foundation for conceptualizing phone attachment as potentially reflecting broader interpersonal attachment patterns, though the two constructs remain distinct.

The application of attachment theory to smartphone use rests on the principle that internal working models generalize beyond interpersonal relationships to inanimate objects and technologies (Bowlby, 1969; Konok et al., 2016). In this context, the smartphone is not merely a tool but can function as a symbolic attachment figure. Its constant, accessible, and responsive presence fulfills core attachment functions: it serves as a safe haven by offering immediate distraction and comfort from distress (e.g., through social media or games), and as a secure base from which to explore the digital and physical world (e.g., via maps, search functions, or communication apps). This dynamic is particularly potent because the device is the primary gateway to - and mediator of—our interpersonal relationships. From a theoretical perspective, for individuals with anxious attachment styles, the phone may become a direct channel for seeking proximity and reassurance, potentially mitigating fears of abandonment through constant connectivity. For those with avoidant attachment styles, the device could serve as a strategic tool to control and distance themselves from the perceived burdens of intimacy. However, these theoretical predictions require empirical testing, as phone attachment and interpersonal attachment styles are distinct constructs that may not directly correspond.

Meta-analytic evidence confirms that insecure interpersonal attachment styles—both anxious and avoidant—are significant predictors of mobile phone addiction (Liang et al., 2021; Lopez-Fernandez, 2017). This body of research supports conceptualizing smartphones as “transitional objects” or “significant others,” particularly for individuals who struggle with in-person intimacy or have experienced childhood trauma (Gritti et al., 2023; Liang et al., 2021; Trub & Barbot, 2016). The development of specialized measurement tools, such as the Young Adult Attachment to Phone Scale (YAPS) and the Mobile Phone Attachment Scale (MPAS), has enabled more precise assessment of these device-focused relational patterns (Bock et al., 2016; Trub & Barbot, 2016).

The need to validate instruments like the YAPS within specific cultural contexts is underscored by the pervasive integration of smartphones into daily life - a trend particularly pronounced in Portugal. Recent data indicate that Portugal has one of the highest smartphone penetration rates in Europe (~ 123.5%) (Kemp, 2024), with over 94% of the population owning a smartphone (European Union, 2023). Furthermore, Portuguese youth report some of the highest daily smartphone usage times in the European Union, averaging over four hours per day (Kemp, 2024; Qustodio, 2024). Despite these indicators of high engagement, there is a distinct lack of validated psychological tools to assess whether this usage constitutes healthy integration or problematic attachment within the Portuguese cultural context. Most research on phone attachment relies on Anglo-Saxon (e.g., Cheever et al., 2014; Elhai et al., 2017) or Asian samples (e.g., Kim et al., 2015; Li et al., 2015), and the generalization of these findings to Southern European cultures—which may differ in social communication styles and family dynamics—remains uncertain.

Therefore, this study aims to address this gap by validating the YAPS for the Portuguese young adult population, providing a culturally sensitive instrument to understand the unique psychological relationship Portuguese young adults have with their phones.

### Attachment to phone

Attachment to one’s phone encompasses emotional, psychological, and behavioral dimensions that extend beyond mere utility or convenience (Hoffner et al. 2016; Konok et al. 2017a). This phenomenon is characterized by significant emotional dependence, manifesting as anxiety, distress, or discomfort when separated from the device—a state often termed “nomophobia” (Konok et al., 2016; Yildirim & Correia, 2015). Behavioral manifestations include frequent checking, compulsive use, and difficulty disengaging from the phone, even during important tasks or social interactions (Clayton et al., 2015; Trub & Barbot, 2016). The phone also serves crucial psychological functions, acting as a tool for emotion regulation, stress management, and social reassurance, sometimes compensating for deficits in real-world interpersonal relationships (Gritti et al., 2023; Liang et al., 2021).

Research consistently links interpersonal attachment patterns to phone attachment. Individuals high in attachment anxiety are more likely to perceive their phones as a “refuge,” experiencing significant discomfort when separated from the device and using it to maintain constant contact with significant others, seeking reassurance and alleviating loneliness (Gritti et al., 2023; Konok et al., 2016; Trub & Barbot, 2016; Wang & Xuang, 2024). In contrast, those with avoidant attachment styles may experience their phones as a “burden,” using them less for emotional support while still developing problematic patterns mediated by loneliness or emotional detachment (Gritti et al., 2023; Konok et al., 2016).

The YAPS operationalizes these theoretical distinctions. Its Refuge dimension captures reliance on the phone for emotional comfort and connection, while the Burden dimension - originally conceptualized by Trub and Barbot (2016) -reflects ambivalence toward the device: experiencing it as both necessary and burdensome. This dimension captures the paradox of phone attachment through items assessing relief when separated (B4), intentional distancing to engage in other activities (B5), and feeling better without the phone (B6). Importantly, these items do not directly measure problematic use; rather, they assess the extent to which individuals experience their relationship with the phone as conflicting or ambivalent. Such ambivalence may reflect avoidant interpersonal tendencies for some individuals, while for others it may indicate healthy boundary-setting or mindful technology use. It is essential to emphasize that these interpretations derive from research on interpersonal attachment patterns; the present study focuses on phone-specific attachment as a distinct construct.

Measurement instruments like the YAPS and MPAS have demonstrated sound psychometric properties, capturing both positive (e.g., usefulness, organization) and negative (e.g., anxious attachment, addiction-like symptoms) aspects of phone attachment (Billieux et al., 2015; Bock et al., 2016; Trub & Barbot, 2016). By distinguishing between healthy and maladaptive forms of phone attachment, these tools offer valuable insights for clinical assessment and intervention (Harris et al., 2020; Trub & Barbot, 2016).

### Rationale for validation in the portuguese population

Although the YAPS and similar scales have demonstrated reliability and validity in their original American contexts, cross-cultural validation is essential before applying these instruments in different linguistic and cultural settings like Portugal (Acar, 2025; Bock et al., 2016; Harris et al., 2020). Such validation extends beyond mere linguistic translation to encompass a thorough assessment of factorial structure, reliability, and construct validity within the new population (Beaton et al., 2000). This rigorous process ensures that the scale accurately reflects local patterns of technology use, social norms, and the meanings attached to mobile phone behaviors—factors that can differ significantly across cultures and influence how phone attachment is experienced and expressed (Gritti et al., 2023).

The need for cultural adaptation is particularly pressing in Portugal, where smartphone use is widespread among young adults. With one of Europe’s highest smartphone penetration rates and daily usage times averaging over four hours (Kemp, 2024; Qustodio, 2024), the country provides a pertinent context for studying phone attachment and its psychological correlates. Yet despite this high engagement, no validated instrument exists to assess whether Portuguese young adults’ phone use constitutes healthy integration or problematic attachment.

Establishing psychometric rigor serves multiple purposes. First, it ensures that the instrument maintains its intended structure and measurement properties, reducing bias and improving research accuracy (Harris et al., 2020). Second, a culturally adapted scale offers clinical and educational utility by enabling early detection of problematic digital behaviors and informing targeted interventions to promote digital well-being and mental health (Bock et al., 2016; Trub & Barbot, 2016). Finally, validated instruments facilitate robust international comparisons, deepening our understanding of both universal and culture-specific aspects of mobile phone use (Gritti et al., 2023).

Accordingly, the present study aims to assess the psychometric properties and factorial structure of the Young Adult Attachment to Phone Scale (YAPS) in a Portuguese sample. This work will contribute to the scientific accuracy of psychological assessments in Portugal while enriching the broader field of research on youth, technology, and mental health.

## Methodology

### Ethical considerations

This study was approved by the Scientific Board of the Universidade Católica Portuguesa (approval number: UCP2024/045). All participants provided informed consent to use and publish the data prior to their participation. Participants were informed about the study’s purpose, the voluntary nature of their participation, their right to withdraw at any time without consequences, and the confidentiality of their data. All data was anonymized and stored securely in compliance with the General Data Protection Regulation (GDPR).

### Participants and procedure

#### Sampling and recruitment

Participants were recruited using a convenience sampling method through university networks and social media platforms. Eligibility criteria included: (1) being between 18 and 35 years of age, (2) currently residing in Portugal, (3) owning a smartphone for at least six months, and (4) having sufficient Portuguese language proficiency to complete the questionnaires.

The sample comprises 648 respondents, with no missing data in most of the analyzed variables. The group is predominantly female (75.8%) and the average age of respondents is 25.07 years (standard deviation of 5.55), with a median age of 23, indicating a generally young population. Regarding educational attainment, 82.7% of the sample attended university.

When it comes to health-related behaviors, approximately 39% of participants report engaging in physical exercise. Among those who do exercise, the average frequency is 3.6 times per week, with a median of 3, suggesting a fairly active subgroup within the sample. Smoking behavior appears prevalent, with 24% of respondents identify as smokers. For those who smoke, the average number of cigarettes consumed daily is approximately 8.34, with a median of 7, suggesting a moderate level of tobacco use among smokers in the sample.

#### Data collection

Data collection took place between December 2024 and February 2025. Participants completed an online survey distributed via Qualtrics, which took approximately 20–25 min. The survey included sociodemographic questions, and the translated version of YAPS. To ensure data quality, attention check items were embedded throughout the survey.

### Instruments

#### Sociodemographic questionnaire

Participants completed a sociodemographic questionnaire that included items on age, gender, education level, employment status, living situation, smartphone usage patterns (e.g., average daily screen time, primary purposes of smartphone use) and physical activity.

#### Young Adult Attachment to Phone Scale (YAPS)

The Young Adult Attachment to Phone Scale (Trub & Barbot, 2016) is a 6-item self-report measure that assesses emotional attachment to mobile phones. The scale consists of two primary dimensions: (1) Refuge - reliance on the phone for emotional regulation and connection, reflecting the phone’s function as a “safe haven” and “secure base”; and (2) Burden - ambivalence toward the phone, capturing the extent to which individuals experience the device as both necessary and burdensome. The Burden dimension includes items that assess relief when separated from the phone (e.g., “Being without my phone gives me a sense of relief”), intentional distancing from the device (e.g., “I intentionally put my phone out of reach to enjoy an activity”), and feeling better when the phone is not present (e.g., “I feel better when I don’t have my phone on me”). It is important to note that these items do not directly measure problematic use; rather, they capture the paradoxical experience of feeling both attached to and burdened by the phone. Items are rated on a 6-point Likert scale ranging from 1 (strongly disagree) to 6 (strongly agree), with higher scores indicating stronger emotional attachment to one’s phone.

Items are rated on a 6-point Likert scale ranging from 1 (strongly disagree) to 6 (strongly agree), with higher scores indicating stronger emotional attachment to one’s phone. None of the YAPS items are reverse-scored. Higher scores on Refuge items indicate greater reliance on the phone for emotional comfort, while higher scores on Burden items indicate greater ambivalence toward the phone (feeling relieved when separated or better without it). The scale is designed such that both dimensions can be endorsed simultaneously, reflecting the paradoxical nature of phone attachment. The original scale demonstrated good internal consistency with Cronbach’s alphas ranging from 0.81 to 0.85 for the subscales (Trub & Barbot, 2016). The YAPS underwent the rigorous translation-back translation procedure to ensure linguistic and cultural appropriateness for the Portuguese population: Two bilingual researchers independently translated the scale from English to Portuguese, and discrepancies were resolved through discussion. A third bilingual researcher back-translated the Portuguese version into English. The back-translated version was compared with the original English version to ensure conceptual equivalence. Adjustments were made until the final Portuguese version was deemed equivalent to the original. Original and Portuguese versions can be found in Table 1.

**Table 1:** Original em Portuguese versions of YAPS

1-Sinto-me ansioso/a ou desconfortável quando não consigo verificar o meu telemóvel.	1-I feel anxious or uncomfortable when I can’t check my phone.
2-Ter o meu telemóvel deixa-me mais seguro/a.	2-Having my phone makes me feel safer.
3-Sinto-me nu/a sem o meu telemóvel.	3-I feel naked without my phone.
4-Estar sem o meu telemóvel dá-me uma sensação de alívio.	4-Being without my phone gives me a sense of relief.
5-Coloco o meu telemóvel intencionalmente fora do meu alcance para desfrutar de uma atividade em que estou envolvido/a.	5-I intentionally put my phone out of reach to enjoy an activity I’m engaged in.
6-Sinto-me melhor quando não tenho o meu telemóvel comigo.	6-I feel better when I don’t have my phone on me.

### Data analysis

All statistical analyses were conducted using R version 4.3.2 (R Core Team, 2023). Descriptive statistics (means, standard deviations, skewness, and kurtosis) were computed using the skimr package (Waring et al., 2026).

Confirmatory factor analysis (CFA) was performed using the lavaan package (Rosseel, 2012) to evaluate the factorial structure of the measurement model. Given the ordinal nature of the YAPS items (6-point Likert scale) and evidence of non-normality (see Table 2), we employed two estimators in a sensitivity analysis framework:

Robust Maximum Likelihood (MLR): Appropriate for continuous data with non-normality (Marôco, 2024; Finney & DiStefano, 2013). This estimator was used in the original analysis and is reported for comparability. Diagonally Weighted Least Squares (DWLS) with robust standard errors: Specifically designed for ordinal data, this estimator treats items as categorical and uses the polychoric correlation matrix (Li, 2016; Rhemtulla et al., 2012). Given the 6-point response scale and the distributional properties of the items (particularly B6 with elevated kurtosis), the DWLS estimator is theoretically more appropriate and is therefore reported as the primary analysis in this manuscript. MLR results are available as supplementary materials.

Model fit was assessed using multiple fit indices: the Comparative Fit Index (CFI; Bentler, 1990), Tucker-Lewis Index (TLI; Tucker & Lewis, 1973), Root Mean Square Error of Approximation (RMSEA; Steiger, 1990), and the Standardized Root Mean Square Residual (SRMR). Following established guidelines (Bentler, 1990; Hu & Bentler, 1999; Marôco, 2021), model fit was considered acceptable when CFI and TLI ≥ 0.90, RMSEA < 0.08, and SRMR < 0.08.

Internal consistency reliability was assessed using Cronbach’s alpha (α) and McDonald’s omega (ω), computed with the semTools package (Jorgensen et al., 2022). Given the ordinal nature of the items, omega was estimated from the DWLS solution using factor loadings from the categorical CFA, which provides more accurate reliability estimates for ordinal scales (Dunn et al., 2014; McDonald, 1999). Reliability was considered acceptable for values ≥ 0.70 (Marôco, 2021; Nunnally & Bernstein, 1994).

Evidence of convergent validity was assessed by examining the Average Variance Extracted (AVE), where values greater than 0.50 indicate adequate convergent validity (Fornell & Larcker, 1981). Discriminant validity was evaluated by ensuring that the square root of the AVE for each factor exceeded the correlation between factors (Fornell & Larcker, 1981).

Measurement invariance across groups (gender and education level) was tested through multi-group CFA using the DWLS estimator. Configural, metric, and scalar invariance were tested hierarchically, following the approach of Cheung and Rensvold (2002) as described in Marôco (2021). Invariance was supported when the change in CFI (ΔCFI) was ≤ 0.010 and the change in RMSEA (ΔRMSEA) was ≤ 0.015 (Chen, 2007; Cheung & Rensvold, 2002).

## Results

### Item descriptives

Table 2 gives the descriptive statistics and histograms for the items of the YAPS. The means ranged from 2.13 (B6) to 3.31 (B2), with standard deviations between 0.67 and 1.35, suggesting some variability in responses. The skewness of the items was generally positive, except for item B2 (–0.23), indicating a slight tendency for respondents to choose lower values on some items. Item B6 exhibited high skewness (1.99) and extreme kurtosis (6.33), suggesting a strong concentration of responses at one point of the scale. However, these extreme skewness and kurtosis values were not impeditive of using CFA with robust maximum likelihood analysis for further psychometric assessment ( DiStefano et al., 2021; Marôco, 2024).

**Table 2:** YAPS’ Item descriptives

Item	Mean (M)	Standard Deviation (SD)	Min	P25	Median (P50)	P75	Max	Skewness (Sk)	Kurtosis (Ku)	Histogram
B1	2.33	1.13	1	1	2	3	5	0.63	–0.38	▆▇▅▂▁
B2	3.31	1.22	1	2	3	4	5	–0.23	–0.91	▂▆▇▇▆
B3	2.46	1.24	1	1	2	3	5	0.48	–0.80	▇▇▆▅▂
B4	2.30	1.07	1	1	2	3	5	0.52	–0.34	▇▇▇▂▁
B5	2.68	1.35	1	1	3	4	5	0.25	–1.17	▇▇▆▆▃
B6	2.13	0.67	1	2	2	2	5	1.99	6.33	▁▇▁▁▁

### Evidence of validity related to internal structure

As conceptualized by Trub and Barbot (2016), the Refuge factor reflects using the phone as a source of emotional comfort and connection, while the Burden factor captures ambivalence toward the phone - experiencing it as both necessary and burdensome. The measurement model demonstrated strong factor loadings across all indicators when estimated using the DWLS estimator, which is appropriate for ordinal data (Table 3). For the ‘Refuge’ factor, items B1 (λ = .761), B2 (λ = .849), and B3 (λ = .800) exhibited high loadings, indicating a robust relationship with the latent construct. Similarly, for the ‘Burden’ factor, items B4 (λ = .645), B5 (λ = .659), and B6 (λ = .541) also showed substantial loadings, suggesting that these items effectively represent the ‘Burden’ construct. All factor loadings were statistically significant (*p* < .001), indicating that each item contributes meaningfully to its respective latent factor.

The measurement model showed excellent fit indices when estimated with DWLS: CFI = 0.990, TLI = 0.980, SRMR = 0.053, RMSEA = 0.069 (90% CI [0.045, 0.094], *p* = .089), indicating that the model adequately represents the data. These results provide strong evidence for the factorial validity of the measurement model in capturing the intended constructs (Table 3).

**Table 3:** Factor loadings for the measurement model (DWLS Estimation)

Latent Factor	Item	λ	SE	Z	Sig
Refuge	B1	0.761	-	-	-
	B2	0.849	0.061	18.36	***
	B3	0.800	0.056	18.69	***
Burden	B4	0.645	-		-
	B5	0.659	0.151	6.78	***
	B6	0.541	0.112	7.50	***

λ = standardized factor loading. Reference indicators (B1 and B4) were fixed to 1.0 for identification, thus no standard errors or z-tests are reported for these parameters. All loadings are statistically significant at *p* < .001

### Internal consistency

Reliability estimates for the YAPS factors were assessed using both Cronbach’s alpha and McDonald’s omega (Table 4). For the Refuge factor, both estimators indicated good internal consistency (α = 0.80, ω = 0.81). For the Burden factor, reliability estimates varied by estimator: Cronbach’s alpha (based on the MLR solution) was 0.54, while McDonald’s omega (based on the DWLS solution with ordinal treatment of items) was 0.74. The omega estimate, which is more appropriate for ordinal data and does not assume tau-equivalence (Dunn et al., 2014; McDonald, 1999), suggests acceptable reliability for the Burden factor. However, the discrepancy between estimators indicates that the Burden factor’s psychometric performance is more sensitive to analytical approach than the Refuge factor, consistent with its fewer items and more varied item content (Table 4). Full MLR-based results are available in Supplementary Materials.

**Table 4:** Internal consistency estimates by estimator

Factor	Cronbach’s α (MLR)	McDonald’s ω (MLR)	McDonald’s ω (DWLS)
Refuge	0.80	0.81	0.83
Burden	0.54	0.59	0.74

### Evidence of convergent and discriminant validity

Convergent validity was assessed using the Average Variance Extracted (AVE) from the DWLS solution. For the Refuge factor, AVE = 0.58, exceeding the recommended threshold of 0.50 and indicating good convergent validity. For the Burden factor, AVE = 0.41, falling slightly below the 0.50 threshold. This suggests that while the Burden items capture meaningful shared variance, there is also substantial unique variance not explained by the latent factor - a common issue with brief scales measuring complex constructs (Fornell & Larcker, 1981).

The correlation between Refuge and Burden factors was *r* = .16 (*p* < .01). The square root of the AVE for Refuge (√AVE = 0.76) exceeded this correlation, and the square root of the AVE for Burden (√AVE = 0.64) also exceeded the correlation, supporting discriminant validity according to the Fornell-Larcker criterion. Thus, despite the lower AVE for Burden, the two factors represent distinct, relatively independent dimensions of phone attachment.

The positive correlation between Refuge and Burden (*r* = .16) warrants explicit commentary. As no items are reverse-scored in the YAPS, this positive association indicates that the two dimensions are not opposites but rather relatively independent aspects of phone attachment. This pattern aligns with the original validation study (Trub & Barbot, 2016), which conceptualized Refuge and Burden as distinct dimensions reflecting the paradox of phone attachment: individuals can simultaneously experience their phone as a source of comfort and connection (Refuge) while also feeling burdened by it or relieved when separated from it (Burden). The small magnitude of the correlation further supports the discriminant validity of the two factors, confirming that they capture related but distinct psychological experiences.

### Evidence of validity based on relations with other variables

To examine patterns of convergent and discriminant validity, we computed correlations between the YAPS factors (Refuge and Burden) and the health-related variables described in the Procedure section: physical exercise and smoking behavior. Given the ordinal nature of the data, Spearman’s rank correlation coefficients were used. Two sets of analyses were conducted: first using dichotomous indicators (whether participants engage in exercise; whether they smoke) and second using frequency indicators among those who reported engaging in each behavior (exercise frequency in times per week; smoking frequency in cigarettes per day).

#### Associations with dichotomous health indicators

Refuge demonstrated a small but statistically significant positive correlation with engaging in physical exercise (*r* = .138, *p* < .001). This finding indicates that individuals who rely on their phones for emotional comfort and connection are slightly more likely to report participating in exercise. While counterintuitive at first glance, this pattern may reflect that socially engaged individuals - including those who use their phones to maintain connections - are also more likely to engage in social or outdoor activities. Alternatively, it may suggest that the Refuge dimension captures a broader tendency toward engagement and social connection rather than purely maladaptive dependence.

In contrast, Burden showed no significant association with exercise participation (*r* = − .075, *p* > .05), supporting the discriminant validity of the two factors. Neither Refuge (*r* = .014, *p* > .05) nor Burden (*r* = .031, *p* > .05) was significantly correlated with smoking status, indicating that phone attachment dimensions are independent of this particular health risk behavior.

#### Associations with frequency indicators

Among participants who reported engaging in physical exercise (*n* = 253), neither Refuge (*r* = .022, *p* > .05) nor Burden (*r* = .036, *p* > .05) was significantly associated with exercise frequency (times per week). Similarly, among participants who reported smoking (*n* = 158), neither Refuge (*r* = − .021, *p* > .05) nor Burden (*r* = − .089, *p* > .05) was significantly correlated with the number of cigarettes smoked per day.

### Measurement invariance for sex and education

Table 5 presents the results of the multi-group invariance analysis for sex and education level, estimated using the DWLS estimator with items treated as ordinal.

**Table 5:** Measurement invariance for sex and education

Modelo	Df	AIC	BIC	χ²	Δχ²	Δdf	*p*(Δχ²)	CFI	RMSEA	ΔCFI	ΔRMSEA
SEX (Male/Female)											
Configural	20	10930.140	11100.150	41.400	—	—	—	0.991	0.058	—	—
Metric	24	10924.820	11076.930	45.600	3.956	4	0.414	0.987	0.063	-0.004	+ 0.005
Scalar	28	10922.650	11056.860	48.100	2.500	4	0.645	0.987	0.062	-0.004	-0.001
Means	30	10923.870	11049.140	48.600	0.561	2	0.755	0.987	0.064	0.000	+ 0.002
EDUCATION (Secondary/University)											
Configural	20	10910.76	11080.76	43.300	—	—	—	0.990	0.060	—	—
Metric	24	10914.16	11066.27	45.800	2.500	4	0.645	0.985	0.062	-0.005	+ 0.002
Scalar	28	10909.80	11044.01	49.500	3.766	4	0.438	0.985	0.061	-0.001	-0.006
Means	30	10922.99	11048.26	59.900	10.352	2	0.006	0.984	0.062	-0.001	+ 0.001

Δχ² values are based on chi-square difference tests for nested models. AIC and BIC are information criteria used for model comparison (lower values indicate better fit). ΔCFI and ΔRMSEA represent changes relative to the previous model. Invariance is supported when ΔCFI ≤ 0.010 and ΔRMSEA ≤ 0.015 (Chen, 2007; Cheung & Rensvold, 2002)

#### Sex (male/female)

The configural model, which allows all parameters to differ across groups, demonstrated excellent fit to the data (CFI = 0.991, RMSEA = 0.058, SRMR = 0.057), indicating that the two-factor structure of the YAPS is equivalent across males and females. This establishes configural invariance.

The metric invariance model, which constrains factor loadings to be equal across groups, showed no significant deterioration in fit compared to the configural model. The change in CFI (ΔCFI = − 0.004) was well within the recommended ≤ 0.010 threshold, and the change in RMSEA (ΔRMSEA = + 0.005) also remained below the ≤ 0.015 criterion. The chi-square difference test was non-significant (Δχ²(4) = 3.96, *p* = .414), further supporting metric invariance. These results indicate that the meaning of the latent factors - Refuge and Burden - is interpreted similarly by males and females.

The scalar invariance model, which constrains item thresholds to be equal across groups, also maintained adequate fit. The change in CFI (ΔCFI = − 0.004) and RMSEA (ΔRMSEA = − 0.001) remained within acceptable limits, and the chi-square difference test was non-significant (Δχ²(4) = 2.50, *p* = .645). This supports scalar invariance, indicating that item thresholds (analogous to intercepts in continuous CFA) are equivalent across sexes. Consequently, latent mean comparisons between males and females are meaningful.

Finally, when latent means were constrained to be equal across groups, the model showed no significant deterioration (ΔCFI = 0.000, ΔRMSEA = + 0.002, Δχ²(2) = 0.56, *p* = .755), indicating no significant differences in latent means between males and females for either Refuge or Burden factors. Full configural, metric, and scalar invariance was supported across sexes, allowing for meaningful comparisons of latent means between males and females.

#### Education (secondary/university)

For education level, the configural model demonstrated good fit (CFI = 0.990, RMSEA = 0.060, SRMR = 0.057), supporting configural invariance and indicating that the two-factor structure holds across participants with secondary and university education.

The metric invariance model, constraining factor loadings to be equal across groups, showed mixed results. The chi-square difference test was non-significant (Δχ²(4) = 2.50, *p* = .645), suggesting that the constraints did not significantly worsen fit. However, the change in CFI (ΔCFI = − 0.005) was within the acceptable ≤ 0.010 threshold, and the change in RMSEA (ΔRMSEA = + 0.002) also remained below the ≤ 0.015 criterion. Based on the recommended guidelines (Chen, 2007; Cheung & Rensvold, 2002), which prioritize CFI and RMSEA changes over the chi-square difference test due to its sensitivity to sample size, metric invariance is supported.

The scalar invariance model, constraining item thresholds across education levels, also maintained adequate fit. The change in CFI (ΔCFI = − 0.001) and RMSEA (ΔRMSEA = − 0.006) remained within acceptable limits, and the chi-square difference test was non-significant (Δχ²(4) = 3.77, *p* = .438). This supports scalar invariance, indicating that item thresholds are equivalent across education levels conditional on the metric model being retained.

When latent means were constrained to be equal across education groups, the model showed a significant deterioration in fit according to the chi-square difference test (Δχ²(2) = 10.35, *p* = .006), although the change in CFI (ΔCFI = − 0.001) remained within acceptable limits. This suggests that latent means differ significantly between secondary and university education groups for at least one of the factors.

Overall, configural, metric, and scalar invariance were supported across education levels, allowing for meaningful comparisons of latent means, though the significant latent mean difference warrants further investigation in future research.

## Discussion

The present study sought to validate the Young Adult Attachment to Phone Scale (YAPS) for the Portuguese young adult population, situating our findings within the broader literature on problematic mobile phone use. The results of the confirmatory factor analysis demonstrated strong factor loadings for the Refuge dimension, which is theoretically consistent with previous research indicating that interpersonal attachment anxiety is a robust predictor of emotional dependence on mobile phones (Gritti et al., 2023; Konok et al., 2016; Trub & Barbot, 2016). The Burden factor, while showing lower internal consistency, still reflected meaningful variance, capturing the ambivalent relationship individuals may have with their phones - feeling both attached to and burdened by the device. This ambivalence is theoretically consistent with findings that avoidant interpersonal attachment may manifest as negative feelings toward phone use, though it can nonetheless contribute to maladaptive patterns under certain conditions (Gritti et al., 2023; Konok et al., 2016). However, as noted by Trub and Barbot (2016), the Burden dimension does not directly measure problematic use or interpersonal attachment avoidance. Instead, it assesses the paradoxical experience of feeling relieved when separated from a device that also serves important functions—a distinction that is critical for interpreting scores on this factor. It is important to emphasize that these interpretations are based on theoretical alignment rather than direct measurement of interpersonal attachment in the present sample.

These results are consistent with the theoretical framework established by Bowlby (1969), which posits that attachment styles developed in early relationships extend into adulthood and may influence interactions with objects and technologies (Konok et al., 2016; Trub & Barbot, 2016). The strong psychometric performance of the Refuge factor provides empirical support for the phone-specific attachment construct, which has been theoretically linked to the concept of smartphones as “transitional objects” or “significant others” (Gritti et al., 2023; Liang et al., 2021; Trub & Barbot, 2016). However, because we did not directly measure interpersonal attachment styles, we cannot conclude that individuals with high Refuge scores in our sample actually possess high interpersonal attachment anxiety. This remains an important question for future research. This is further supported by meta-analytic evidence demonstrating that insecure attachment styles, both anxious and avoidant, are significant predictors of mobile phone addiction (Liang et al., 2021; Panova & Carbonell, 2018; Zhang et al., 2022).

The convergent and discriminant validity results, as demonstrated by the Fornell-Larcker criteria, confirm that the Refuge and Burden dimensions capture distinct aspects of phone attachment, consistent with the multidimensional conceptualization proposed in earlier studies (Bock et al., 2016; Trub & Barbot, 2016). The scale’s ability to distinguish between these dimensions is particularly important for both clinical assessment and intervention. If future research establishes links between phone attachment and interpersonal attachment patterns, individuals with high Refuge scores may benefit from strategies aimed at fostering secure attachment and alternative sources of social support, while those with higher Burden scores may require interventions focused on reducing digital stress and promoting healthier boundaries (Harris et al., 2020; Trub & Barbot, 2016). At present, the YAPS should be viewed as a measure of phone-specific emotional attachment, with its clinical implications for broader attachment patterns requiring further investigation.

Evidence of validity based on relations with other variables provided preliminary support for the distinctiveness of the two YAPS factors. Refuge showed a small but significant positive correlation with exercise participation, indicating that individuals who use their phones as a source of emotional comfort are slightly more likely to engage in physical activity. While this finding may appear counterintuitive at first - given that problematic phone use is often associated with sedentary behavior - it aligns with research suggesting that phone attachment is not uniformly maladaptive. Trub and Barbot (2016) conceptualized the Refuge dimension as reflecting the phone’s function as a “safe haven” and “secure base,” which may facilitate rather than hinder real-world engagement for some individuals. In this sense, individuals who are socially connected via their phones may also be more oriented toward social and outdoor activities, including exercise. In contrast, Burden showed no association with exercise participation, supporting the discriminant validity of the two factors and reinforcing that ambivalence toward the phone represents a distinct psychological experience.

Neither Refuge nor Burden were significantly associated with smoking status or with the frequency of exercise or smoking among those who engaged in these behaviors. These null findings are consistent with expectations, as phone attachment is conceptually distinct from health risk behaviors such as smoking. The absence of strong correlations with these external variables suggests that the YAPS captures a unique construct not reducible to general health behaviors or lifestyle factors. This pattern aligns with the original YAPS validation study, which emphasized that phone attachment represents a specific psychological relationship with technology rather than a proxy for broader behavioral patterns (Trub & Barbot, 2016).

An important finding of this study is the positive correlation between Refuge and Burden (*r* = .16), which may initially seem counterintuitive. Readers might expect that feeling safer with the phone (Refuge) would be inversely related to feeling better without it (Burden). However, as clarified in the Results section, no items are reverse-scored, and this positive correlation reflects the theoretically grounded paradox of phone attachment articulated by Trub and Barbot (2016). The YAPS was deliberately designed to capture the ambivalent relationship many individuals have with their smartphones—the same device that serves as a source of comfort and connection can also be experienced as intrusive or overwhelming. This duality is consistent with contemporary understandings of human-technology relationships, which recognize that emotional bonds with devices are often complex and multifaceted rather than unidimensional (Gritti et al., 2023; Konok et al., 2016). The small magnitude of the correlation further confirms that Refuge and Burden are distinct constructs, supporting the two-factor structure of the YAPS in the Portuguese context.

An important consideration in interpreting the Burden factor concerns the content of its items, particularly item B5 (“I intentionally put my phone out of reach to enjoy an activity”). As the reviewer noted, this item could reflect either avoidant tendencies toward social connection through the phone or, alternatively, healthy self-regulation and mindful technology use. This dual interpretation highlights the complexity of measuring phone-related ambivalence: intentional distancing from the phone may be maladaptive for some individuals (e.g., those using the phone to avoid intimacy) but adaptive for others (e.g., those practicing digital wellness). Recent research has begun to explore these nuances, suggesting that the relationship between avoidant attachment and phone use is complex and potentially bidirectional (Gritti et al., 2023; Sun & Miller, 2023). For some individuals with avoidant tendencies, the phone may serve as a tool to control social availability and create distance from others; for others, putting the phone away may represent a healthy boundary rather than avoidance. Future qualitative research should explore how Portuguese young adults interpret these items, particularly whether they view intentional phone distancing as a positive self-regulatory strategy or as a reflection of discomfort with connection.

The measurement invariance analyses revealed that the YAPS performs equivalently across genders, supporting its use for direct comparisons between males and females, as recommended in cross-cultural validation guidelines (Beaton et al., 2000; Gjersing et al., 2010). For education levels, configural and scalar invariance were established, although some caution is warranted in interpreting mean differences due to partial support for metric invariance. This finding is consistent with previous cross-cultural adaptation studies, which emphasize the importance of ensuring both structural and measurement equivalence when applying psychological instruments in new contexts (Beaton et al., 2000; Lopez-Fernandez et al., 2017).

The lower internal consistency observed for the Burden factor suggests the need for further refinement, and the conceptual ambiguity of items such as B5 (“I intentionally put my phone out of reach”) underscores the importance of qualitative research to explore how Portuguese young adults interpret items related to phone-related stress, ambivalence, and intentional distancing from the device. Understanding whether respondents view these behaviors as positive self-regulation or as indicators of discomfort with connection is essential for refining the measurement of phone attachment in this cultural context. This is in line with recommendations from Harris et al. (2020) and Bock et al. (2016), who highlight the importance of continuous scale adaptation and validation in diverse populations.

This study presents some limitations. Its cross-sectional design does not allow for conclusions about causality between attachment styles and phone use (Trub & Barbot, 2016). Reliance on self-report measures may introduce bias, as participants might under- or overestimate their behaviors (Harris et al., 2020). The Burden factor showed lower internal consistency, suggesting that further refinement or additional items may be needed for this dimension in the Portuguese context (Trub & Barbot, 2016). The psychometric properties of the Burden factor were sensitive to estimator choice. While DWLS estimation - appropriate for ordinal data - yielded acceptable reliability (ω = 0.74), the AVE fell below conventional thresholds, and the factor’s performance was weaker than the original validation sample. This suggests that the Burden dimension may require further refinement for the Portuguese context, potentially through additional items or qualitative exploration of how young adults experience phone-related ambivalence. In particular, item B5 (“I intentionally put my phone out of reach to enjoy an activity”) may be interpreted in multiple ways - as healthy boundary-setting, as mindful technology use, or as avoidance of connection. Future research should employ cognitive interviewing or qualitative methods to understand how Portuguese young adults interpret this item and whether it functions as intended across different subgroups. Researchers using the Portuguese YAPS should interpret Burden scores with caution and consider supplementing with other measures of problematic phone use when possible.

This study did not assess participants’ interpersonal attachment styles. While we interpret our findings through the lens of attachment theory - given that the YAPS was explicitly developed to measure phone-specific attachment as conceptually distinct from interpersonal attachment (Trub & Barbot, 2016) - we cannot draw direct conclusions about the relationship between YAPS scores and participants’ actual interpersonal attachment patterns. The theoretical connections drawn between phone attachment and attachment anxiety or avoidance should be considered speculative until empirically tested in studies that include direct measures of adult attachment (e.g., Experiences in Close Relationships Scale; ECR). Future research should examine whether and how the Refuge and Burden dimensions correspond to anxious and avoidant interpersonal attachment styles in Portuguese young adults.

The use of convenience sampling, mainly among university students and with overrepresentation of female participants (75.8%), limits the generalizability of the findings to all Portuguese young adults (Hussain et al., 2017). Finally, although measurement invariance was supported across gender, only partial support was found for education level, so group comparisons should be interpreted with caution (Cheung & Rensvold, 2002).

Future research should use longitudinal designs to clarify how attachment to phones develops over time and whether it predicts later problematic use (Trub & Barbot, 2016; Wang & Xuang, 2024). Studies should also explore additional mediators and moderators, such as loneliness, self-regulation, and mental health symptoms, to better understand the mechanisms linking attachment styles to phone dependence (Elhai et al., 2020; Wang & Xuang, 2024). Improving the reliability of the Burden dimension and validating the scale in broader and more diverse samples, including non-students, will strengthen its utility (Harris et al., 2020; Trub & Barbot, 2016). Finally, combining self-report with objective measures of phone use could provide a more accurate picture of digital behaviors (Harris et al., 2020).

Overall, the findings of this study support the validity and reliability of the Portuguese version of the YAPS, while also highlighting areas for further research and refinement. The results underscore the importance of culturally sensitive assessment tools for understanding the complex, multidimensional nature of phone attachment and its implications for mental health and social functioning among young adults (Gritti et al., 2023; Harris et al., 2020; Trub & Barbot, 2016).

## Conclusion

This study makes a significant contribution to the field by successfully validating the Young Adult Attachment to Phone Scale (YAPS) for the Portuguese young adult population, thereby providing a culturally and linguistically appropriate tool for assessing phone attachment in this specific context. The research adds to knowledge in several key ways. First, it confirms the cross-cultural applicability of the theoretical link between attachment theory and problematic mobile phone use. The strong psychometric performance of the Refuge dimension solidifies the understanding of the smartphone as a “transitional object” for individuals with high attachment anxiety, offering empirical support within a new cultural setting.

Second, the study provides nuanced insights into the less-explored Burden dimension. While its lower internal consistency indicates a need for future refinement, its confirmation is theoretically consistent with findings that avoidant interpersonal attachment can manifest as ambivalence toward phone use. However, the Burden dimension captures a broader construct of phone-related ambivalence that may include both maladaptive avoidance and healthy boundary-setting, highlighting the need for continued refinement and qualitative exploration of how individuals interpret items such as intentionally distancing themselves from their phones.

Third, the demonstration of full measurement invariance across gender is a critical advancement, as it allows for confident and direct comparisons of YAPS scores between males and females in future Portuguese research. The partial invariance across education levels also provides valuable cautions and directions for further study.

Finally, by providing a validated instrument, this study enables more accurate clinical assessment and tailored interventions in Portugal. Practitioners can now distinguish between individuals who use their phone as a refuge (needing strategies for secure attachment) and those who see it as a burden (needing help with digital boundaries), thereby enhancing mental health support for young adults.

This paper not only offers a validated tool for the Portuguese context but also deepens the theoretical understanding of phone attachment, underscores the importance of cultural validation, and provides a foundation for more precise research and clinical practice.

## Supplementary Information

Supplementary Material 1.

Supplementary Material 2.

## Data Availability

Data is available on reasonable request.
