# Supplementary material for: Assessing mobile phone attachment: a psychometric study in the Portuguese young adult population
Source: Psicol Reflex Crit. 2026 Jun 10;39:28. doi: 10.1186/s41155-026-00400-6 (PMC13473055; doi:10.1186/s41155-026-00400-6)
Supplement: Supplementary file 1 [file 41155_2026_400_MOESM1_ESM.docx]

**Title page**

**Title**

**Assessing Mobile Phone Attachment: A Psychometric Study in the Portuguese Young Adult Population**

**Running head**

**Mobile Phone Attachment in Portuguese Adults**

Ângela Leite^1*^, João Marôco^2^

^1^Centro de Estudos Filosóficos e Humanísticos, Universidade Católica Portuguesa, Praça da Faculdade, Braga, Portugal. <https://orcid.org/0000-0003-0560-1756>; [aleite@ucp.pt](mailto:aleite@ucp.pt); *corresponding author

^2^ Intrepid Lab, ECEO, Universidade Lusófona & CETRAD, UTAD. Lisboa, Portugal, Lisboa. Portugal. <https://orcid.org/0000-0001-9214-5378>; [joao.maroco@ulusofona.pt](mailto:joao.maroco@ulusofona.pt)

**Abstract**

Background: The Young Adult Attachment to Phone Scale (YAPS) is a 6-item self-report tool that measures emotional attachment to smartphones. It gauges two core aspects: using the phone for emotional comfort and connection and experiencing anxiety during separation from it. Aim: The present paper aims to assess the psychometric properties of the YAPS for the Portuguese young adult population. Methods: Based on 648 participants, psychometric properties were rigorously assessed, with CFA confirming the hypothesized structure, excellent reliability (α/ω ≥ .70), and strong evidence of convergent and discriminant validity. Multi-group CFA further established measurement invariance across groups. Results: The model structure fit the data well across multiple fit criteria. Internal consistency was excellent for the Refuge factor but moderately lower for Burden, suggesting room for refinement in the latter. Both factors met thresholds for convergent and discriminant validity (capturing distinct constructs), with clear differentiation from each other. The scale performed equally well across genders, allowing direct comparisons between males and females. For education levels, the structure remained consistent, though some metric differences emerged, requiring cautious interpretation of group comparisons. Latent mean differences indicated varying scores across education levels, highlighting demographic variations in phone attachment patterns. Conclusion: Attachment to mobile phones is a complex, multidimensional phenomenon with significant implications for mental health and social functioning, especially among young adults. Validating culturally sensitive assessment tools like the YAPS in the Portuguese context is a critical step toward advancing research, prevention, and intervention efforts in the digital age.

*Keywords*: Attachment, Problematic Behavior Related to Mobile Phone, Burden, Refuge, Young Adult Attachment to Phone Scale

**Statements and Declarations**

**Competing Interests**

The authors declare no financial or non-financial conflicts of interest.

**Funding**

This study received no funding.

**Authors Contributions**

Ângela Leite and João Marôco made substantial contributions to the study conception and design. João Marôco performed material preparation and analysis. The first draft of the manuscript was written by Ângela Leite and both authors commented on previous versions of the manuscript. Also, both authors read and approved the final manuscript.

**Ethics approval**

This study was approved by the Ethics Committee of the Universidade Católica Portuguesa (Conselho Científico, reference UCP2024/045) and conducted in accordance with the Declaration of Helsinki.

**Consent to participate**

Informed consent was obtained from all individual participants included in the study.

**Consent to publish**

Consent to publish was obtained from all individual participants included in the study.

**Data transparency**

Data is available on reasonable request.

**Large Language Models**

During the preparation of this work, the authors used CHATGPT to help in the writing process to improve the readability and language of the manuscript. After using this tool, the authors reviewed and edited the content as needed and took full responsibility for the content of the published article. Thus, the authors declare they have not used any AI services to generate or edit any part of the manuscript or data.

**Authors' information (optional)**

Ângela Leite has a degree in Philosophy and Psychology, and a PhD in Biomedical Sciences. She is an assistant professor at the Catholic University of Portugal and a researcher at the Center for Philosophical and Humanistic Studies. Her publications are mostly in the area of Health Psychology, especially in the relationship between medicine and psychology. She is currently investigating the relation between human behavior and climate change.

João Marôco is a professor of statistics and research methods at Universidade Lusófona. His research interest focus on university students' burnout and engagement, psychometrics and statistical modelling. He is a Top 2% world researcher in the 2022's Stanford University Ranking having published more than 450 research papers, four books and two dozen book chapters (Google Scholar H = 76).
