# Supplementary material for: Assessing mobile phone attachment: a psychometric study in the Portuguese young adult population
Source: Psicol Reflex Crit. 2026 Jun 10;39:28. doi: 10.1186/s41155-026-00400-6 (PMC13473055; doi:10.1186/s41155-026-00400-6)
Supplement: Supplementary file 2 [file 41155_2026_400_MOESM2_ESM.docx]

**Supplementary materials**

Analysis performed with Robust Maximum Likelihood (MLR)

**Evidence of validity related to internal structure**

The measurement model demonstrates strong factor loadings across all indicators (Table 3). For the 'Refuge' factor, items B1 (β = 0.761), B2 (β = 0.849), and B3 (β = 0.800) exhibit high loadings, indicating a robust relationship with the latent construct. Similarly, for the 'Burden' factor, items B4 (β = 0.645), B5 (β = 0.659), and B6 (β = 0.541) also show substantial loadings, suggesting that these items effectively represent the 'Burden' construct. All factor loadings are statistically significant (p < 0.001), indicating that each item contributes meaningfully to its respective latent factor. The measurement model showed good fit indices (CFI = 0.983, TLI = 0.968, NFI = 0.979, SRMR = 0.053, RMSEA = 0.077, 90% CI ]0.054, 0.102[, p = 0.029), indicating that the model adequately represents the data. These results present evidence sources related to the factorial validity of the measurement model in capturing the intended constructs (Table 3).

Table 3
*Factor Loadings for the Measurement Model*

| **Latent Factor** | **Item** | **Beta** | **SE** | **Z** | **Sig** |
| --- | --- | --- | --- | --- | --- |
| Refuge | B1 | 0.761 | 0.087 | 13.44 | *** |
|  | B2 | 0.849 | 0.159 | 10.09 | *** |
|  | B3 | 0.800 | 0.107 | 12.50 | *** |
| Burden | B4 | 0.645 | 0.102 | 8.30 | *** |
|  | B5 | 0.659 | 0.110 | 7.95 | *** |
|  | B6 | 0.541 | 0.079 | 8.14 | *** |

**Internal consistency**

The reliability estimates for the first-order model were assessed using Cronbach’s alpha and McDonald's omega. For the Refuge factor, Cronbach’s alpha was 0.804, while the McDonald's omega was 0.814. For the Burden factor, Cronbach’s alpha was 0.540, while McDonald's omega for the Burden factor was 0.592. These results indicate good internal consistency for the Refuge factor, with values consistent across the different reliability measures. In contrast, the Burden factor exhibited lower reliability, with alpha and omega below the generally accepted threshold of 0.7.

**Evidence of convergent and discriminant validity**

The Fornell-Larcker matrix revealed that the square root of the Average Variance Extracted (AVE) for "Refuge" is 0.81, which exceeds the recommended threshold of 0.5, indicating good convergent validity. For "Burden," the AVE is 0.622, which also meets the criterion for convergent validity. The correlations between "Refuge" and "Burden" (0.136) are lower than the square root of the AVE for each factor, supporting the discriminant validity of the measurement model. Thus, both factors exhibit appropriate convergent and discriminant validity according to the Fornell-Larcker criteria (Fornell & Larcker, 1981) (Table 4).

Table 4

*Fornell and Larcker’s AVE and scales correlation*

|  | **Refuge** | **Burden** |
| --- | --- | --- |
| **Refuge** | 0.81 |  |
| **Burden** | 0.136 | 0.622 |

*Note*: The values on the main diagonal represent the square root of the Average Variance Extracted (AVE), while the off-diagonal values are the correlations between the factors.

**Measurement Invariance for Sex and Education**

Table 5 presents the results of the invariance analysis for Sex and Education. Regarding Sex, the configural model showed good fit (CFI = .967, RMSEA = .072), indicating that the factorial structure is equivalent across males and females. Imposing equality constraints on factor loadings (metric invariance) slightly improved model fit (ΔCFI = .002, ΔRMSEA = −.009), and the chi-square difference test was not significant (Δχ²(4) = 2.77, p = .598), supporting metric invariance. Further imposing equality on intercepts (scalar invariance) also maintained adequate fit (ΔCFI = −.002, ΔRMSEA = −.003, p = .177), indicating scalar invariance. When latent means were constrained to be equal, the difference was also non-significant (Δχ²(2) = 5.03, p = .081, ΔCFI = −.004, ΔRMSEA =-0.001), suggesting no significant latent mean differences between sexes. The data suggests that full configural, metric, and scalar invariance was supported by sex, allowing for meaningful comparisons of latent means across groups.

Regarding Education, configural model fit the data well (CFI = .973, RMSEA = .065), supporting a common factorial structure across education levels. However, the metric model yielded a significant decrease in fit (Δχ²(4) = 16.99, p = .002), although ΔCFI = −.009, was within the acceptable (<0.01) threshold partially supporting metric invariance. Despite this, the scalar model did not worsen model fit compared to the metric model (ΔCFI = .000, Δχ²(4) = 3.77, p = .439), indicating scalar invariance conditional on the metric model being retained. The test for equality of latent means showed a significant difference (Δχ²(2) = 10.35, p = .006), indicating that latent means differ by education level. The results support that configural and scalar invariance were met, while metric invariance was supported only by ΔCFI. Therefore, comparisons of latent means across education levels should be interpreted with caution.

Table 5

*Measurement invariance for Sex and Education*

| **Modelo** | **Df** | **AIC** | **BIC** | **χ²** | **Δχ²** | **Δdf** | **p(Δχ²)** | **CFI** | **RMSEA** | **ΔCFI** | **ΔRMSEA** |
| --- | --- | --- | --- | --- | --- | --- | --- | --- | --- | --- | --- |
| SEX (Male/Female) | | | | | | | | | | | |
| Configural | 16 | 10930.14 | 11100.15 | 42.885 |  |  |  | 0.967 | 0.072 | 0.000 | 0.000 |
| Metric | 20 | 10924.82 | 11076.93 | 45.567 | 2.766 | 4 | 0.598 | 0.969 | 0.063 | 0.002 | -0.009 |
| Scalar | 24 | 10922.65 | 11056.86 | 51.393 | 6.314 | 4 | 0.177 | 0.966 | 0.059 | -0.002 | -0.003 |
| Means | 26 | 10923.87 | 11049.14 | 56.619 | 5.028 | 2 | 0.081 | 0.966 | 0.059 | 0.004 | -0.001 |
| EDUCATION (Secondary/University) | | | | | | | | | | | |
| Configural | 16 | 10910.76 | 11080.76 | 37.956 |  |  |  | 0.973 | 0.065 | 0.000 | 0.000 |
| Metric | 20 | 10914.16 | 11066.27 | 49.354 | 16.985 | 4 | 0.002 | 0.964 | 0.067 | -0.009 | 0.002 |
| Scalar | 24 | 10909.80 | 11044.01 | 52.995 | 3.766 | 4 | 0.439 | 0.964 | 0.061 | 0.000 | -0.006 |
| Means | 26 | 10922.99 | 11048.26 | 70.192 | 10.352 | 2 | 0.006 | 0.964 | 0.061 | 0.019 | -0.011 |

Note: **Δχ²** are corrected with Santorra-Bentler correction for skewness and kurtosis.
